# Draft Genome Sequence of Bacillus cereus ET31, Isolated from an Extreme Environment

**DOI:** 10.1128/MRA.01061-19

**Published:** 2019-11-07

**Authors:** Panagiota Stathopoulou, Sofia Nikolaki, Maria Lanara, George Tsiamis

**Affiliations:** a Department of Environmental Engineering, University of Patras, Agrinio, Greece; Loyola University Chicago

## Abstract

Bacillus cereus is a Gram‐positive, widely distributed bacterium that has a high level of metabolite production. Here, we report the draft genome sequence of a B. cereus strain exhibiting high and diverse hydrolytic potential that was isolated from glacial water samples from Svalbard, Norway.

## ANNOUNCEMENT

Bacterial communities associated with glaciers found all over the world are being increasingly studied for their potential biotechnological applications (1). The Svalbard archipelago (74° to 81° N, 10° to 35° E) comprises an area of 62,248 km^2^ with more than 2,000 glaciers, and ice covers 60% of its surface (2). As a result, the draft genome sequence of Bacillus cereus ET31, which was isolated from glacier sediment from Svalbard Islands and has a high and diverse hydrolytic potential, is reported here.

Bacillus cereus is a Gram-positive, rod-shaped, aerobic or facultatively anaerobic, motile, beta-hemolytic, spore-forming bacterium that is widely distributed environmentally due to its ability to form thermoresistant endospores and biofilms and to grow over a broad temperature range (3, 4). Moreover, B. cereus has a high level of production of diverse metabolites, including bacteriocins, autolysins, and enzymes, particularly proteases (5–9).

This article reports the draft genome sequences of B. cereus strain ET31, isolated from glacial water in Svalbard, Norway (10). Bacteria were isolated on Antarctic bacterial medium (ABM) agar (0.5% [wt/vol] peptone, 0.2% [wt/vol] yeast extract, and 1.5% [wt/vol] agar, pH 7.0) at 12°C under aerobic conditions. Bacillus cereus ET31 was identified among 300 bacterial isolates obtained from glacier water from geologically and hydrologically diverse regions of southern Svalbard. Genomic DNA extraction from one culture, grown as described previously, was performed according to Haught et al. (11). The DNA was sent to MicrobesNG, a next-generation sequencing (NGS) service provider in the United Kingdom, for 16S rRNA and whole-genome sequencing. DNA was quantified in triplicates with the Quant-iT double-stranded DNA (dsDNA) high-sensitivity (HS) assay (Invitrogen) in an Ependorff AF2200 plate reader. The genomic DNA library was prepared using the Nextera XT library prep kit (Illumina, San Diego, CA), following the manufacturer’s protocol with the following modifications: 2 ng of DNA was used as input instead of 1 ng, and PCR elongation time was increased to 1 min from 30 s. DNA quantification and library preparation were carried out on a Hamilton Microlab Star automated liquid-handling system. The library was quantified using the Kapa Biosystems library quantification kit for Illumina on a Roche LightCycler 96 quantitative PCR (qPCR) machine. The library was sequenced with 52-fold coverage on the Illumina HiSeq platform using a 250-bp paired-end protocol, producing 714,745 reads. Reads were adapter trimmed using Trimmomatic v. 0.30 with a sliding window quality cutoff of Q15 (12). FastQC v. 0.11.8 with default parameters was used to check the quality of the validated reads (13). Reads were *de novo* assembled into 34 contigs using SPAdes v. 3.7 (14). The quality of the assembly was evaluated with Quast (15). Taxonomic assignment of the reads was calculated using Kraken (16), and Prokka v. 1.11 (17) was used for genome annotation. Protein function was predicted by performing sequence similarity queries against the UniProt Knowledgebase (UniProtKB) (18).

The assembled draft genome had a length of 5,875,600 bp, with a GC content of 34.9%. The largest contig was 2,021,752 bp long, the *L*_50_ was 2, and the *N*_50_ value equaled 1,600,101 bp. The majority of reads (94.07%) were classified into the genus Bacillus, and 59.55% were placed in the B. cereus group. The draft genome contained 5,859 protein coding sequences, with 2,535 of them identified as encoding hypothetical proteins, 99 tRNA sequences, 13 sequences identified as 5S rRNAs, and 1 as a 16S rRNA.

### Data availability.

The whole-genome shotgun project of B. cereus ET31 has been deposited at DDBJ/ENA/GenBank under accession number VSSM00000000, BioProject number PRJNA560934, and BioSample number SAMN12603168. The raw sequencing reads were deposited to the Sequence Read Archive (SRA) under accession number SRR9997721. The version described in this paper is the first version, VSSM01000000.
